# Supplementary material for: AI-enhanced reconstruction of the 12-lead electrocardiogram via 3-leads with accurate clinical assessment
Source: NPJ Digit Med. 2024 Aug 1;7:201. doi: 10.1038/s41746-024-01193-7 (PMC11294561; doi:10.1038/s41746-024-01193-7)
Supplement: Supplementary file 1 — Supplemental Material [file 41746_2024_1193_MOESM1_ESM.pdf]

SUPPLEMENTARY INFORMATION

Supplementary Note 1 – Learning architecture

The learning algorithms used in this paper were developed by using Pytorch, a well-known machine learning framework that can be integrated with both Python and C++. The full code of the learning algorithms, written in Python, is available at: https://github.com/scripps-research/ecg_reconstruction.

The reconstruction architecture is organized into three consecutive sections, as overviewed in the main manuscript, which specified the characteristics of the residual convolutional neural network (ResCNN). All the inner layers of the architecture are followed by a rectified linear unit (RELU) function, that enables better gradient propagation. The final layer does not present any activation function, so the output is given by the linear combination of the neurons. The convolutional layers are associated with a 17 x C_{in} x C_{out} kernel, where C_{in} and C_{out} are the number of input and output channels, respectively. The padding and the stride operations were tuned to ensure that the input dimensionality (in terms of sample number) was maintained fixed across the entire architecture.

During the training of the learning architecture, we considered Adam as the algorithm for tuning the weights and biases of the ResCNN layers, setting the learning rate to 10^{-6}, the Nesterov momentum to 0.9, and the weight decay to 10^{-3} respectively. Both the learning algorithms were trained for a maximum of 200 epochs, considering an early stopping approach to prevent overfitting in the training set, and setting the batch size to 16.

Supplementary Note 2 – Training the reconstruction algorithm

To train and evaluate the reconstruction algorithm, we considered the Mean Squared Error (MSE) and the coefficient of determination (R2) between the original precordial leads and those generated by the learning architecture. For a given ECG, x and y denoted the leads taken as input by the reconstruction algorithm and the precordial leads that the algorithm must reconstruct, respectively. The mean square error (MSE) between the original precordial leads and those generated by the reconstruction algorithm is then given by

$$MSE(\,y) = \sum_{i\,\in(V1,V2,V3,V4,V5,V6)} \frac{1}{6}\sum_{n=1}^{2500} \frac{|\hat{y}(i,n) - \,y(i,n)|^2}{2500}, \tag{1}$$

where $|\cdot|$ is the absolute value function, $y(i,n)$ represents the nth sample of lead i, while y and $\hat{y}$ are the original and reconstructed precordial leads, respectively.

The coefficient of determination (R2) is a standardized version of MSE since it is independent of the scale of the reconstructed signals. In our scenario, we define R2 between the original and reconstructed precordial leads as

$$R2(y) = \sum_{i\,\in(V1,V2,V3,V4,V5,V6)} \frac{1}{6}\sum_{n=1}^{2500} \left(1 - \frac{|\hat{y}(i,n) - \,y(i,n)|^2}{|y(i,n) - \,\bar{y}(i)|^2}\right), \tag{2}$$

where $\bar{y}(i) = \sum_{n=1}^{2500} y(i,n)/2500$ is the sample mean of lead i. During the training phase, the opposite of R2 was considered as the loss function of our reconstruction: therefore, minimizing the loss function is equivalent to maximizing the expectation of R2 on the training data.

During the training phase, the opposite of R2 was considered as the loss function of our reconstruction.

Supplementary Note 3 – Training the classification algorithm

As the loss function for the classification algorithm, we considered the cross entropy (CE) between the true labels of the data and those predicted by the learning architecture. For a given ECG, we denoted by x the 12 leads of the ECG, and by z a Boolean variable equal to 1 in case the signal shows evidence of an acute MI or 0 without evidence of an acute MI. The CE between z and the algorithm output $\hat{z}$ is then given by

$$CE(x,z) = z \cdot \log(\hat{z}) + (1 - z) \cdot \log(1 - \hat{z})\,. \tag{3}$$

Before evaluating the accuracy of the classification algorithm, we carried out an additional training phase to refine the working of the reconstruction algorithm. The goal was to combine the reconstruction and classification architectures to emphasize the features necessary to detect electrocardiographic evidence consistent with acute MI. Hence, the reconstruction algorithm was retrained with the dual aim of providing an accurate reconstruction of the ECG signal, as well as providing the best input for the classification algorithm. This goal was achieved by designing a more advanced loss function, depending on both the CE between the true and predicted data label and the R2 between the original and reconstructed precordial leads.

When combining the reconstruction and classification algorithms, we considered a different loss of function depending on both the R2 between the original and reconstructed leads, and the CE between the true and predicted labels. For a given ECG, we denoted by x any combination of input leads, by y the original precordial leads of the signal, by z the Boolean variable indicating the presence of acute MI, and by $w(x)$ the set of 12 leads obtained substituting the original precordial

leads with those generated by the reconstruction algorithm. Hence, the reconstruction algorithm was trained to minimize

$$L(x, y, z) = -\alpha \cdot R2(x, y) + (1 - \alpha) \cdot CE(w(x), z), \quad (4)$$

where α is a scalar parameter between 0 and 1. This loss function encourages the reconstruction algorithm to improve the classification performance while keeping a high reconstruction quality. By tuning the value of α, it is possible to prioritize the minimization of the mathematical distance between the original and reconstructed signals (given by the R2 term), or the maximization of the detection accuracy (given by the CE term). In the paper's results, we set $\alpha = 0$, seeking the maximization of the detection performance.

Supplementary Note 4 – Explainable analysis

To understand which lead is considered more significant by our approach for reconstructing a 12-lead ECG, we implemented Shapley additive explanations (SHAP), one of the most common explainable artificial intelligence (XAI) methods, to analyze the reconstruction algorithm. Given a general learning model, SHAP makes it possible to associate each input feature with a value denoting the feature's importance in generating the output. In our case, SHAP allowed us to determine the importance of each sample of the input leads for reconstructing a 12-lead ECG. Particularly, we considered I+II+V3 as the input configuration of the learning architecture and lead V1 as the reconstruction target: hence, SHAP assigned higher importance to the input samples with a larger impact on the reconstruction of lead V1.

Running the SHAP algorithm over the whole test set, we estimated the probability distribution of the sample importance for each input lead of the I+II+V3 reconstruction architecture. The results denoted that the mean of the sample importance is associated with a value of 0.10 for lead I, 0.04 for lead V3, and 0.07 for lead II. (Supplementary Figure 3)

This implies that, on average, lead I contains the most significant information for reconstructing V1. On the other side, as expected, lead V3 is, on some specific cases, even more important than lead I for the reconstruction. Overall, the results emphasized how adding a precordial lead to the input is fundamental for improving the reconstruction accuracy.

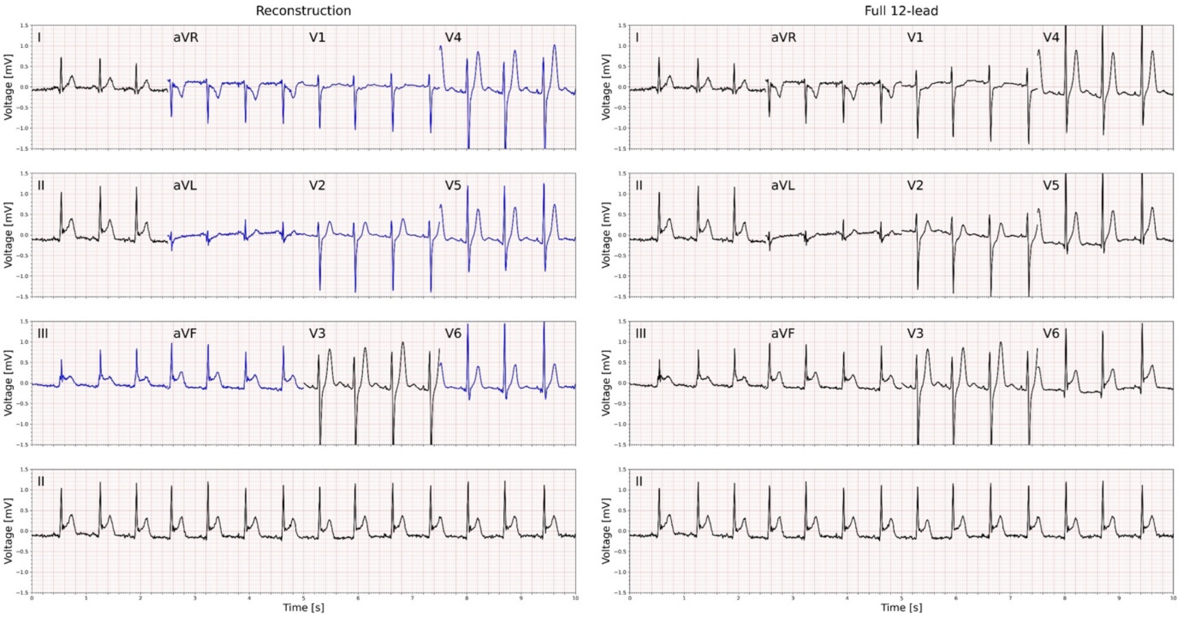
Reconstruction
Full 12-lead
I
aVR
V1
V4
II
aVL
V2
V5
III
aVF
V3
V6
II
Voltage [mV]
Time [s]

(a)

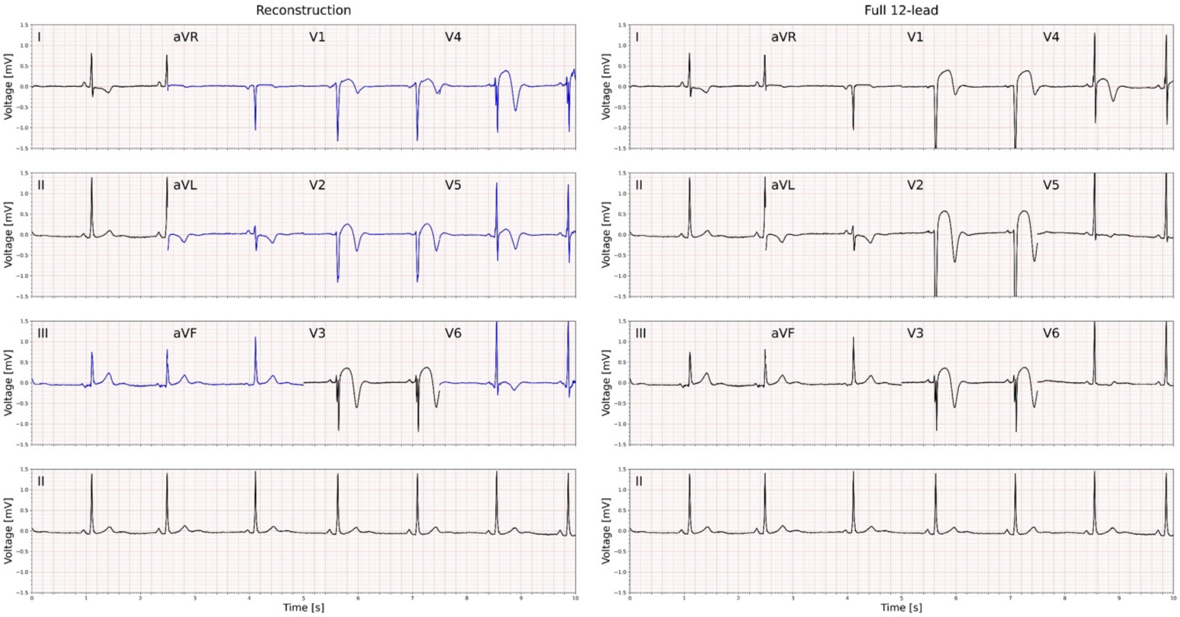
Reconstruction
Full 12-lead
I
aVR
V1
V4
II
aVL
V2
V5
III
aVF
V3
V6
II
Voltage [mV]
Time [s]

(b)

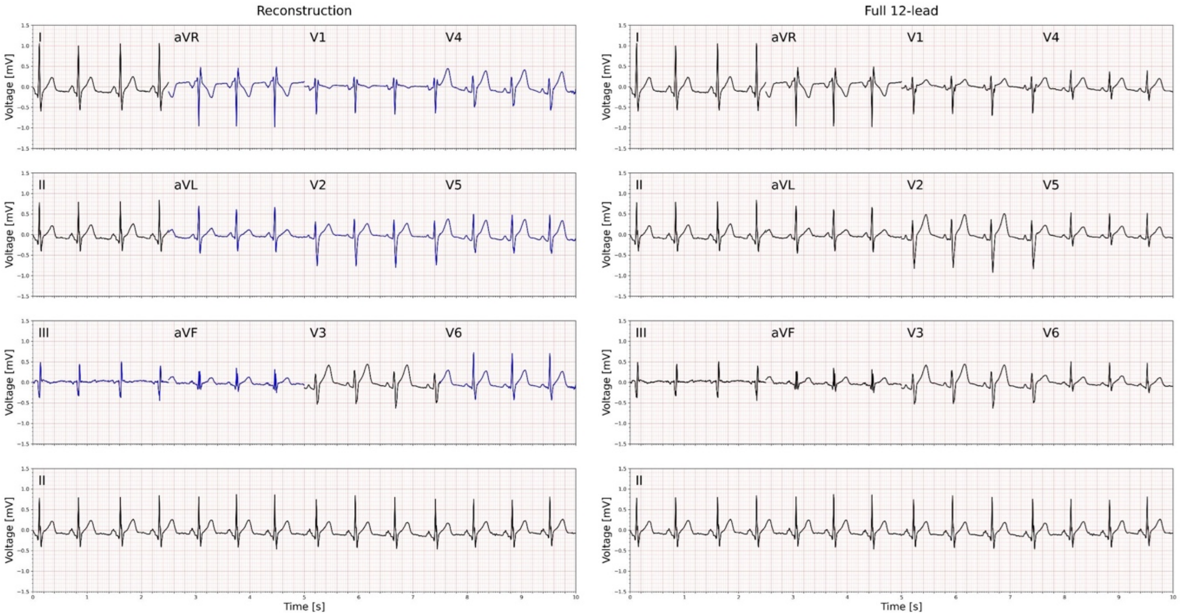

(c)

Supplementary Figure 1. **Example of ECG reconstruction.**

The figure represents a reconstructed ECG. In panel (a) an ECG presenting inferior ST-segment elevated myocardial infarction (STEMI), in panel (b) an ECG presenting anterior STEMI, and in panel (c) an ECG in normal sinus rhythm.

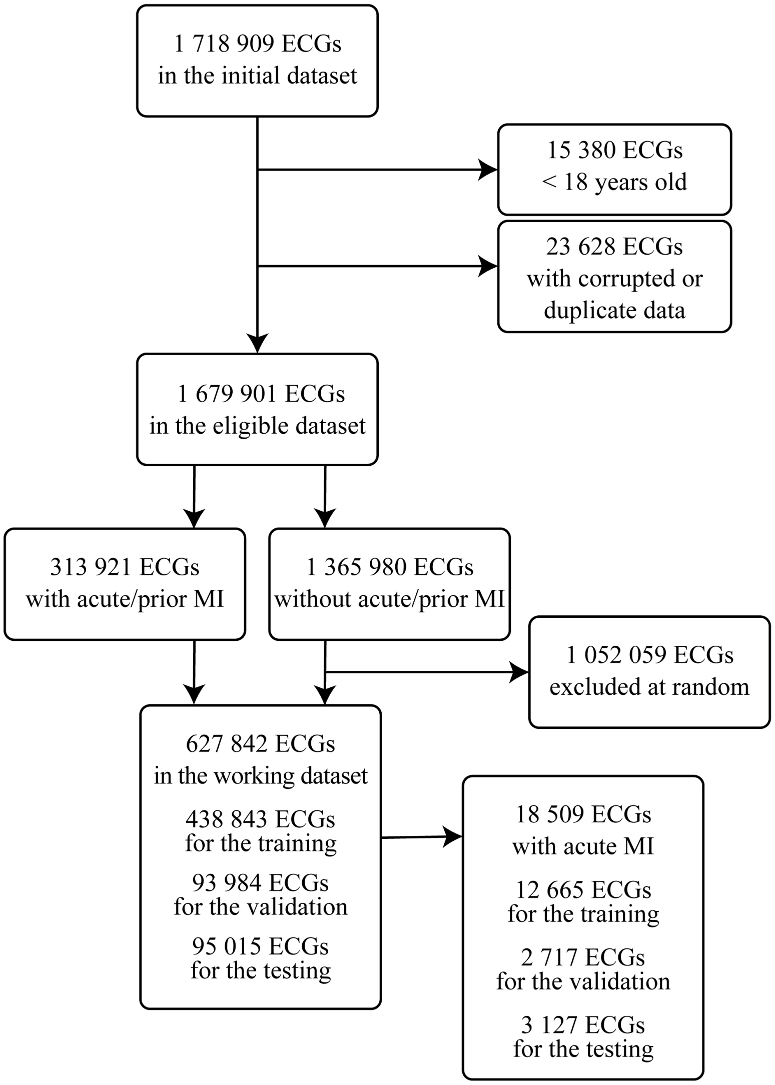

Supplementary Figure 2. **Dataset organization and exclusion criteria.**

From the initial dataset, we excluded all the duplicate ECGs (i.e., identical ECGs recorded multiple times), the ECGs associated with individuals younger than 18 years old, and the ECGs presenting corrupted data. From the eligible dataset, we selected all the ECGs associated with evidence of acute or prior infarcts (313,921 ECGs), and we randomly selected an equal number of ECGs without infarct evidence. The working dataset (627,842 ECGs) was divided into three mutually exclusive subsets, including the ECGs for the training, validation, and testing phases, respectively. In the working dataset, the data associated with acute MI (18,509 ECGs) were considered positive elements for the classification algorithm.

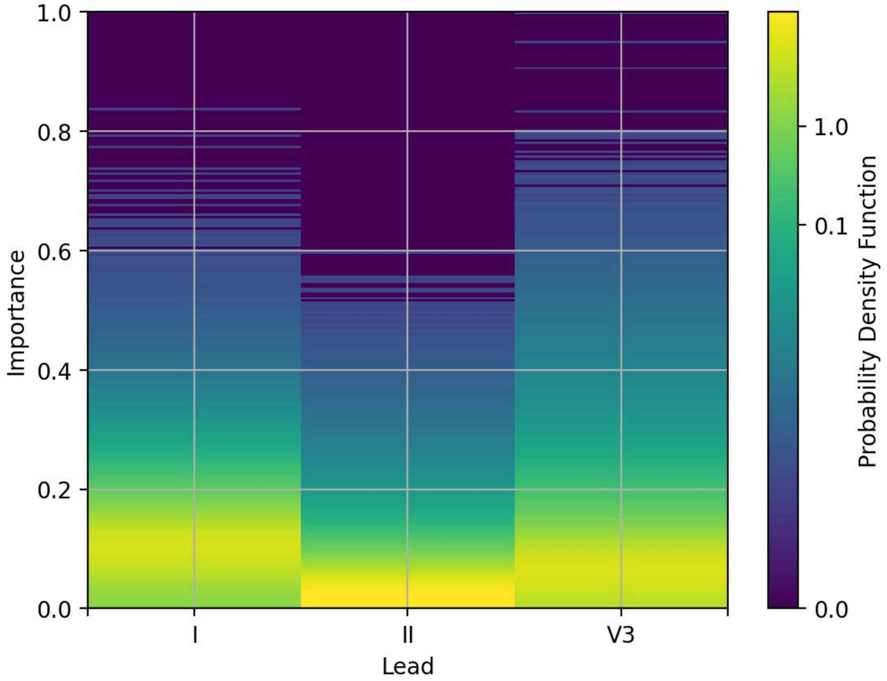

Supplement Figure 3**. Probability distribution of the sample importance for the I+II+V3 reconstruction algorithm.**

The figure depicts the probability distribution of the sample importance, computed by the SHAP algorithm, for each input lead of the I+II+V3 reconstruction architecture; the importance values were normalized within the $[0, 1]$ range, where 0 and 1 are the minimum and maximum importance, respectively; a lighter value denotes a higher probability for a sample to be associated with a specific importance level.

		Number of elements in the overall dataset		R2 [%] in test set (average ± confidence interval)		MSE [mV^2] in test set (average ± confidence interval)		Sensitivity (for specificity = 90.00%) in test set (average ± confidence interval)		
		Number of individuals	Number of ECGs	Using I+II	Using I+II+V3	Using I+II	Using I+II+V3	Using I+II	Using I+II+V3	Using 12-leads
	Total	11234	18509	43.8 ± 1.4	69.9 ± 0.9	0.0366 ± 0.0016	0.0171 ± 0.0009	77.7 ± 1.4	86.7 ± 1.2	87.5 ± 1.2
Acute myocardial infarct (positive data)	Anterior MI	1213	1519	46.7 ± 2.4	72.3 ± 1.6	0.0417 ± 0.0046	0.0193 ± 0.0030	57.1 ± 5.8	77.0 ± 4,9	88.3 ± 3.8
	Septal MI	59	69	52.9 ± 8.2	75.8 ± 4.1	0.0189 ± 0.0083	0.0089 ± 0.0031	53.9 ± 27.1	46.2 ± 27.1	46.2 ± 27.1
	Lateral MI	894	1080	38.22 ± 5.4	65.3 ± 4.1	0.0419 ± 0.0091	0.0239 ± 0.0077	80.2 ± 7.1	81.5 ± 6.0	80.2 ± 6.1
	Anteroseptal MI	784	974	40.44 ± 3.3	71.0 ± 2.6	0.0467 ± 0.0087	0.0221 ± 0.0077	71.0 ± 7.0	87.7 ± 5.1	88.9 ± 4.8
	Anterolateral MI	929	1168	39.63 ± 3.4	69.1 ± 2.4	0.0581 ± 0.0086	0.0248 ± 0.0041	85.0 ± 4.9	91.5 ± 3.8	92.0 ± 3.8
	Inferolateral MI	623	755	46.99 ± 5.1	68.6 ± 3.5	0.0304 ± 0.0088	0.0164 ± 0.0054	98.0 ± 2.3	97.9 ± 2.3	96.5 ± 3.0
	Inferior or posterior MI	3985	5963	44.40 ± 2.0	69.1 ± 1.8	0.0321 ± 0.0027	0.0155 ± 0.0017	90.0 ± 1.8	91.4 ± 1.7	90.1 ± 1.8
	Localization unspecified MI	5458	7570	44.0 ± 2.6	70.45 ± 1.4	0.0353 ± 0.0023	0.0162 ± 0.0012	75.9 ± 2.4	84.9 ± 2.0	85.4 ± 2.0

Supplementary Table 1. **Reconstruction and classification performance according to the acute MI localization.**

The table describes the system performance associated with different classes of acute MI, depending on the localization of the injury. For each class, the table reports the total number of individuals and the total number of ECGs. The reconstruction performance is assessed in terms of coefficient of determination (R2) and mean squared error (MSE). The classification performance is given by fixing the specificity to 0.90 and evaluating the corresponding sensitivity. The confidence interval was computed considering a confidence level of 95%.

Sinus rhythm	Normal sinus rhythm	Conduction disorders	First degree AV block
	Sinus arrhythmia		Second degree AV block
	Sinus bradycardia		Third degree AV block
	Sinus tachycardia		Sinoatrial block
Atrial arrhythmia	Atrial arrhythmia		Right bundle branch block
	Atrial bradycardia		Left bundle branch block
	Atrial tachycardia		Left anterior fascicular block
	Atrial flutter		Left posterior fascicular block
	Atrial fibrillation		Bi-fascicular block
	Atrial arrhythmia		Tri-fascicular block
	Atrial bigeminy		Intraventricular conduction delay
	Atrial trigeminy		Right ventricular conduction delay
	Premature atrial complexes		Left ventricular conduction delay
	Wolff Parkinson White		Wide QRS
Ventricular arrhythmia	Ventricular arrhythmia		Short PR
	Ventricular bradycardia		Long QT
	Ventricular tachycardia	Repolarization abnormality	ST segment elevation
	Ventricular flutter		ST segment depression
	Ventricular fibrillation		ST Segment abnormality
	Ventricular bigeminy		T wave inversion
	Ventricular trigeminy		T wave flattening
	Premature ventricular complexes		T wave abnormality
	Brugada syndrome		Early repolarization
Other arrhythmias	Premature idioventricular complexes	Cardiac hypertrophy	Left atrial hypertrophy
	Premature junctional complexes		Right atrial hypertrophy
	Premature fusion complexes		Left ventricular hypertrophy
	Electrical Pacemaker		Right ventricular hypertrophy
		Axis deviation	Right axis deviation
Myocardial infarct			Left axis deviation
Myocardial ischemia			Extreme axis deviation

Supplementary Table 2. **Labels used for describing the electrocardiographic features.**
