# Supplementary material for: AI-enhanced reconstruction of the 12-lead electrocardiogram via 3-leads with accurate clinical assessment
Source: NPJ Digit Med. 2024 Aug 1;7:201. doi: 10.1038/s41746-024-01193-7 (PMC11294561; doi:10.1038/s41746-024-01193-7)
Supplement: Supplementary file 2 — Reporting summary [file 41746_2024_1193_MOESM2_ESM.pdf]

nature portfolio

Corresponding author(s): Giorgio Quer
Last updated by author(s): Jun 25, 2024

Reporting Summary

Nature Portfolio wishes to improve the reproducibility of the work that we publish. This form provides structure for consistency and transparency in reporting. For further information on Nature Portfolio policies, see our Editorial Policies and the Editorial Policy Checklist.

Statistics

For all statistical analyses, confirm that the following items are present in the figure legend, table legend, main text, or Methods section.

n/a	Confirmed	
☐	☒	The exact sample size (*n*) for each experimental group/condition, given as a discrete number and unit of measurement
☐	☒	A statement on whether measurements were taken from distinct samples or whether the same sample was measured repeatedly
☐	☒	The statistical test(s) used AND whether they are one- or two-sided<br>*Only common tests should be described solely by name; describe more complex techniques in the Methods section.*
☐	☒	A description of all covariates tested
☒	☐	A description of any assumptions or corrections, such as tests of normality and adjustment for multiple comparisons
☐	☒	A full description of the statistical parameters including central tendency (e.g. means) or other basic estimates (e.g. regression coefficient) AND variation (e.g. standard deviation) or associated estimates of uncertainty (e.g. confidence intervals)
☐	☒	For null hypothesis testing, the test statistic (e.g. *F*, *t*, *r*) with confidence intervals, effect sizes, degrees of freedom and *P* value noted<br>*Give P values as exact values whenever suitable.*
☒	☐	For Bayesian analysis, information on the choice of priors and Markov chain Monte Carlo settings
☒	☐	For hierarchical and complex designs, identification of the appropriate level for tests and full reporting of outcomes
☒	☐	Estimates of effect sizes (e.g. Cohen's *d*, Pearson's *r*), indicating how they were calculated

Our web collection on statistics for biologists contains articles on many of the points above.

Software and code

Policy information about availability of computer code

Data collection	The retrospective data used in this study were collected through the Scripps Health GE MUSE system before the beginning of the study.
Data analysis	The analyses performed in this study were carried out via an ad-hoc code, written in Python 3.10, which is available at: https://github.com/scripps-research/ecg_reconstruction. For training the learning models, as well as for data processing and visualization, the following Python libraries have been employed: PyTorch, NumPy, pandas, SciPy, seaborn, and Matplotlib.

For manuscripts utilizing custom algorithms or software that are central to the research but not yet described in published literature, software must be made available to editors and reviewers. We strongly encourage code deposition in a community repository (e.g. GitHub). See the Nature Portfolio guidelines for submitting code & software for further information.

Data

Policy information about availability of data

All manuscripts must include a data availability statement. This statement should provide the following information, where applicable:

- Accession codes, unique identifiers, or web links for publicly available datasets
- A description of any restrictions on data availability
- For clinical datasets or third party data, please ensure that the statement adheres to our policy

This study is retrospective, and it did not generate any new data. The data used in this study were obtained from Scripps Health and are subject to restrictions that

prevent public sharing. Detailed information regarding the data source and acquisition process is provided in the manuscript. Due to the terms of the data use agreement and compliance with ethical and legal requirements, the data cannot be made openly available.

Research involving human participants, their data, or biological material

Policy information about studies with human participants or human data. See also policy information about sex, gender (identity/presentation), and sexual orientation and race, ethnicity and racism.

Reporting on sex and gender	Each element of the dataset used in this is study is associated with the sex (Male or Female) of the individual from whom the element was recorded, as specified by the Scripps Health GE MUSE system. No information about the gender and/or sexual orientation of the individuals is reported in the dataset. The results of the study were analyzed as a function of the sex.
Reporting on race, ethnicity, or other socially relevant groupings	Each element of the dataset is associated with the race (White or Non-White) of the individual from whom the element was recorded, as specified by the Scripps Health GE MUSE system. No information about the ethnicity of the individuals is reported in the study. The results of the study were analyzed as a function of the race.
Population characteristics	The main clinical and demographic covariates used in the study are the following: age, sex, race, ECG rhythm, ECG morphological features (conduction disorders, repolarization abnormalities, cardiac hypertrophy, axis deviation), and signs of past/ongoing coronary heart diseases (ischemia, infarction).
Recruitment	This study is retrospective and is based on a pre-existent ECG dataset collected in the Scripps Health facilities from 2008 to 2019. Hence, no recruitment process has occurred.
Ethics oversight	The protocol for this study was reviewed by the Scripps Office for the Protection of Research Subjects and determined to be exempt from formal committee review (IRB-20-7504).

Note that full information on the approval of the study protocol must also be provided in the manuscript.

Field-specific reporting

Please select the one below that is the best fit for your research. If you are not sure, read the appropriate sections before making your selection.

☒ Life sciences ☐ Behavioural & social sciences ☐ Ecological, evolutionary & environmental sciences

For a reference copy of the document with all sections, see nature.com/documents/nr-reporting-summary-flat.pdf

Life sciences study design

All studies must disclose on these points even when the disclosure is negative.

Sample size	The dataset used in the study was defined by choosing all the available ECGs associated with the past/present signs of myocardial infarctions and an equal number of signals without such characteristics. The second group of signals was chosen randomly from the Scripps Health dataset, without any exclusion/inclusion criteria. The total size of the working dataset is 627,842.
Data exclusions	We excluded all the ECGs associated with individuals younger than 18 years old, all the duplicate ECGs, and all the ECGs presenting signal errors and/or missing demographic information.
Replication	All the proposed metrics in our study are deterministic, ensuring that the results are fully reproducible. The entire processing pipeline has been thoroughly tested.
Randomization	The working dataset used in the study was divided into three mutually exclusive subsets, including the data for the training, validation, and testing of the learning models. Such a division was performed randomly, ensuring that all the ECGs recorded from the same patient were inserted in the same subset (so that the data of the same patient cannot be used for both training and validating/testing purpose). The detailed procedure for this allocation process can be found in the manuscript.
Blinding	The working dataset organization in different groups was performed by a computer program.

Reporting for specific materials, systems and methods

We require information from authors about some types of materials, experimental systems and methods used in many studies. Here, indicate whether each material, system or method listed is relevant to your study. If you are not sure if a list item applies to your research, read the appropriate section before selecting a response.

Materials & experimental systems

n/a	Involved in the study
☒	☐ Antibodies
☒	☐ Eukaryotic cell lines
☒	☐ Palaeontology and archaeology
☒	☐ Animals and other organisms
☒	☐ Clinical data
☒	☐ Dual use research of concern
☒	☐ Plants

Methods

n/a	Involved in the study
☒	☐ ChIP-seq
☒	☐ Flow cytometry
☒	☐ MRI-based neuroimaging

Plants

Seed stocks	*Report on the source of all seed stocks or other plant material used. If applicable, state the seed stock centre and catalogue number. If plant specimens were collected from the field, describe the collection location, date and sampling procedures.*
Novel plant genotypes	*Describe the methods by which all novel plant genotypes were produced. This includes those generated by transgenic approaches, gene editing, chemical/radiation-based mutagenesis and hybridization. For transgenic lines, describe the transformation method, the number of independent lines analyzed and the generation upon which experiments were performed. For gene-edited lines, describe the editor used, the endogenous sequence targeted for editing, the targeting guide RNA sequence (if applicable) and how the editor was applied.*
Authentication	*Describe any authentication procedures for each seed stock used or novel genotype generated. Describe any experiments used to assess the effect of a mutation and, where applicable, how potential secondary effects (e.g. second site T-DNA insertions, mosiacism, off-target gene editing) were examined.*
